# The seed development of a mycoheterotrophic orchid, *Cyrtosia javanica* Blume

**DOI:** 10.1186/s40529-014-0044-8

**Published:** 2014-05-30

**Authors:** Chih-Kai Yang, Yung-I Lee

**Affiliations:** 1 The Experimental Forest, College of Bio-Resources and Agriculture, National Taiwan University, 12 Chienshan Rd., Sec. 1, Chushan Township, Nantou 55750 Taiwan; 2 Department of Life Science, National Taiwan Normal University, 88 Tingchow Rd., Sec. 4, Taipei, 11677 Taiwan; 3 Biology Department, National Museum of Natural Science, No 1, Kuan-Chien Rd, Taichung, Taiwan; 4 Department of Life Sciences, National Chung Hsing University, Taichung, 40227 Taiwan

**Keywords:** Embryo, Mycoheterotrophic orchid, Seed coat, Vanilloid orchids

## Abstract

**Background:**

*Cyrtosia javanica* is a rare, mycoheterotrophic vanilloid orchid native to the bamboo forest in central Taiwan. Like some vanilloid orchids, the seeds of *C. javanica* are hard and difficult to germinate in vitro. A better understanding of the embryology would provide insights in the propagation and conservation of this rare species.

**Results:**

Based on the histological and histochemical studies, we observed some remarkable features in developing seeds of *C. javanica*. First, the developing embryos without a structurally defined suspensor; Second, the chalazal accessory cells have densely stained cytoplasms that are different from the adjacent cells of seed coat; Third, the multiple layers of seed coat with the lignified in the outermost cell layer of the outer seed coat.

**Conclusions:**

In *C. javanica*, the large and heavy seeds embedded in fresh fruits may adapt to the dispersal strategy. The hard seeds with lignified outer seed coat could provide a rigid protection during seed dispersal but also cause coat-imposed dormancy. This study provides insights in the seed coat structure and the hints of seed treatment methods.

**Electronic supplementary material:**

The online version of this article (doi:10.1186/s40529-014-0044-8) contains supplementary material, which is available to authorized users.

## Background

A seed is a small embryonic plant enclosed in a protective covering called the seed coat (Fenner and Thompson [2005]). As compared to most flowering plants, the structure of orchid seeds is minute and simple (Dressler [1993]). The orchid seeds generally lack a well-defined endosperm and contain a globular-shaped embryo covering by the thin layers of seed coats (Arditti [1992]). Although the macroscopic appearances of various orchid seeds are similar, the orchid seeds are highly diverse owing to their seed coats (Clements and Molvray [1999]). The morphological variability in seed coats of orchids may relate to the dispersal strategy and seed dormancy. The seeds of *Vanilla* species are hard and black, and embedded in the fresh and non-dehiscent fruits that are adapted to bird-dispersed (Cameron and Chase [1998]; Rodolphe et al. [2011]). These characteristics, i.e. the hard seed body and the fresh fruit are unique that could be observed in the basal genera of orchid family, such as *Apostasia*, *Selenipedium* and *Vanilla* (Nishimura and Tamura [1993]; Nishimura and Yukawa [2010]).

*Cyrtosia* is a fully mycoheterotrophic genus belonging to the subfamily Vanilloideae of Orchidaceae (Cameron [2009]). The species of *Cyrtosia* have a broad distribution from Taiwan, Southern China, Indo-China and tropic Asia areas. The plants of *Cyrtosia javanica* are leafless with large underground rhizomes. In Taiwan, *C. javanica* was first described at Xitou in 1995, and then could not be found for a long time (Su [2000]). More recently, it was rediscovered nearby, and the population size is small and restricted in a bamboo forest (Yang et al. [2010]). In our investigations on *C. javanica*, the aboveground shoots only appear for flowering and fruit setting within a short period (approximately one month). As the fruits matured, the fresh fruits contain several relatively large seeds with brown color that are likely to be bat-dispersed (our preliminary observations). Besides, the in vitro germination of this species is complicated (our unpublished data). Until now, the information about the developmental biology of seeds in *C. javanica* is still limited. A better understanding of the seed biology and mycoheterotrophic relationship would provide insights in the conservation of rare orchid species (Dixon et al. [2003]). The objectives of this study were to document the anatomical events in seed development of *C. javanica* from fertilization to seed maturity, and to detail the formation of the seed coat. The information presented in this study may provide the background knowledge for the seed germination of this mycoheterotrophic species.

## Methods

### Plant materials

**Figure 1:**
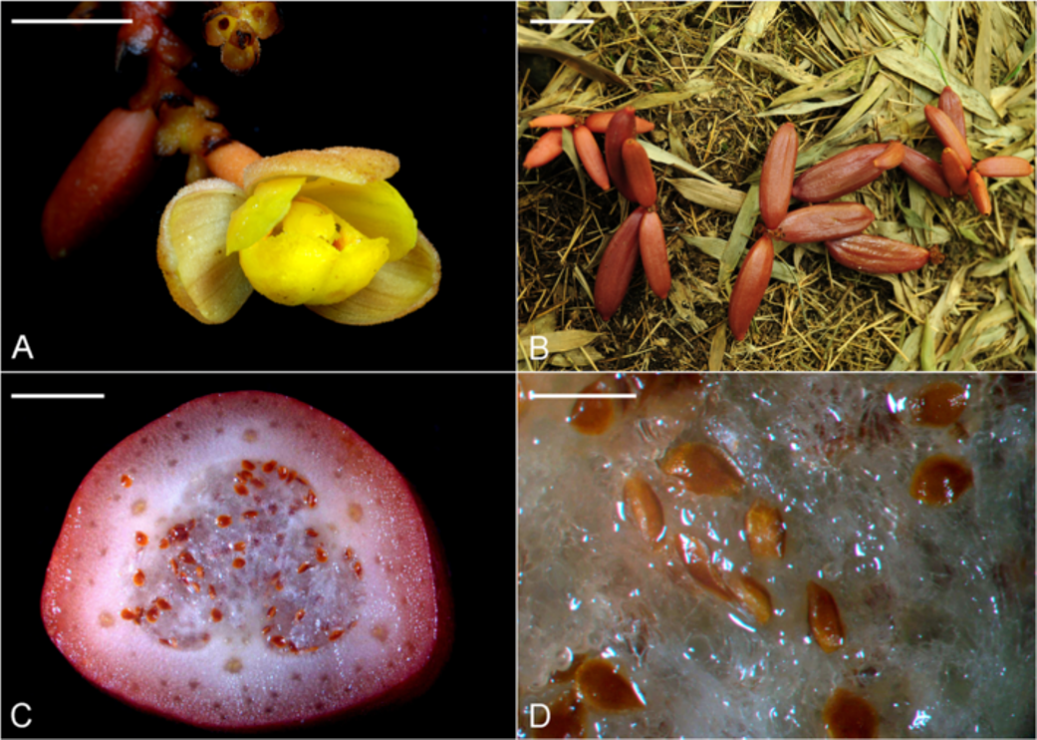
**The flowers and developing fruits of** ***Cyrtosia javanica*** **. (A)** The flower of *Cyrtosia javanica*. Scale bar = 2 cm. **(B)** The developing fruits of *Cyrtosia javanica* in the natural habitat. Scale bar = 4 cm. **(C)** The cross section of a maturing fruit. Scale bar = 5 mm. **(D)** The seed has turned brown. Scale bar = 0.8 mm.

Developing capsules of *C. javanica* were collected from a natural population in a bamboo forest located at Xitou, Nantou, Taiwan. Anthesis usually occurred in early June, each year (Figure 1A). After flowering, developing capsules of different stages were collected at regular intervals for histological and histochemical studies (Figure 1B-D). Approximately 20 developing fruits were gathered for this study.

### Light microscopy and histochemical studies

Transverse sections, approximately 3 mm thick of developing fruits were fixed in 2.5% glutaraldehyde and 1.6% paraformaldehyde buffered with 0.05 M phosphate buffer, pH 6.8, for 3 days at 4°C. After fixation, the sections were dehydrated in methyl cellosolve (BDH Chemicals) for 24 hours, followed by two changes of 100% ethanol for 24 hours each at 4°C. The samples were infiltrated gradually (3:1. 1:1, and 1:3 100% ethanol: Historesin, 24 hours each) with Historesin (Leica Canada, Markham, Ontario), followed by two changes of pure Historesin. The tissues were then embedded according to Yeung ([1999]). Longitudinal sections of 3 μm thick were obtained using Ralph knives on a Reichert-Jung 2040 Autocut rotary microtome. Sections were stained with the periodic acid-Schiff’s (PAS) reaction for total insoluble carbohydrates and counter-stained with either 0.05% (w/v) toluidine blue O (TBO) in benzoate buffer for general histology or 1% (w/v) amido black 10B in 7% acetic acid for protein (Yeung [1984]). The sections were viewed and the images were captured digitally using a CCD camera attached to a light microscope (Axioskop 2, Carl Zeiss AG, Germany). The Historesin embedded tissues were stained with 1 μg ml^−1^ of Nile red (Sigma Chemical Co., St. Louis, Mo.), following the procedures of Yeung et al. ([1996]). The fluorescence pattern was examined using an epifluorescence microscope (Axioskop 2, Carl Zeiss AG) equipped with the Zeiss filter set 15 (546/12 nm excitation filter and 590 emission barrier filter), and the images were captured digitally using a CCD camera.

## Results

### Embryo development

**Figure 2:**
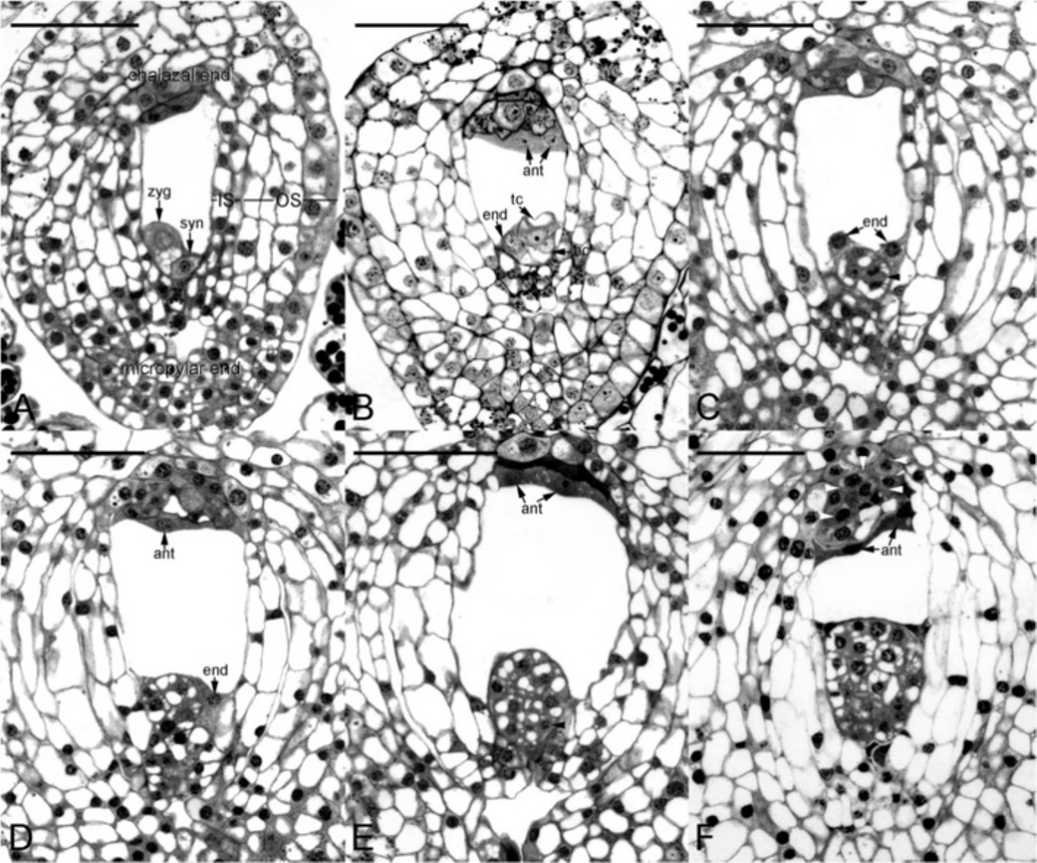
**Early embryo development of** ***Cyrtosia javanica*** **. (A)** The zygote has a dense cytoplasm and a prominent nucleus located at the chalazal end. **(B)** The first cell division of the zygote results in the formation of a terminal cell and a basal cell. In this species, the endosperm fails to develop, and one endosperm nucleus could be observed to stay beside the 2-celled embryo at the micropylar end in this light micrograph. **(C)** Light micrograph showing a further cell division (arrowhead) occurs at the basal cell of a proembryo. Two endosperm nuclei do not further develop and will soon degenerate. **(D)** Light micrograph showing a proembryo without a prominent suspensor differentiated. The antipodal cells are located at the chalazal end and densely stained. **(E)** An anticlinal division (arrowhead) occurs in the outmost cell layer, resulting in the formation of the globular-shape embryo. **(F)** Light micrograph showing an early globular embryo without a distinct suspensor structure. The chalazal accessory cells are distinguished from the adjacent cells of seed coat by their densely stained cytoplasm (arrowheads). Abbreviations: ant = antipodal cells; bc = basal cell; end = endosperm nuclei; IS = inner seed coat; OS = outer seed coat; syn = synergid; tc = terminal cell; zyg = zygote. Scale bar = 100 μm.

**Figure 3:**
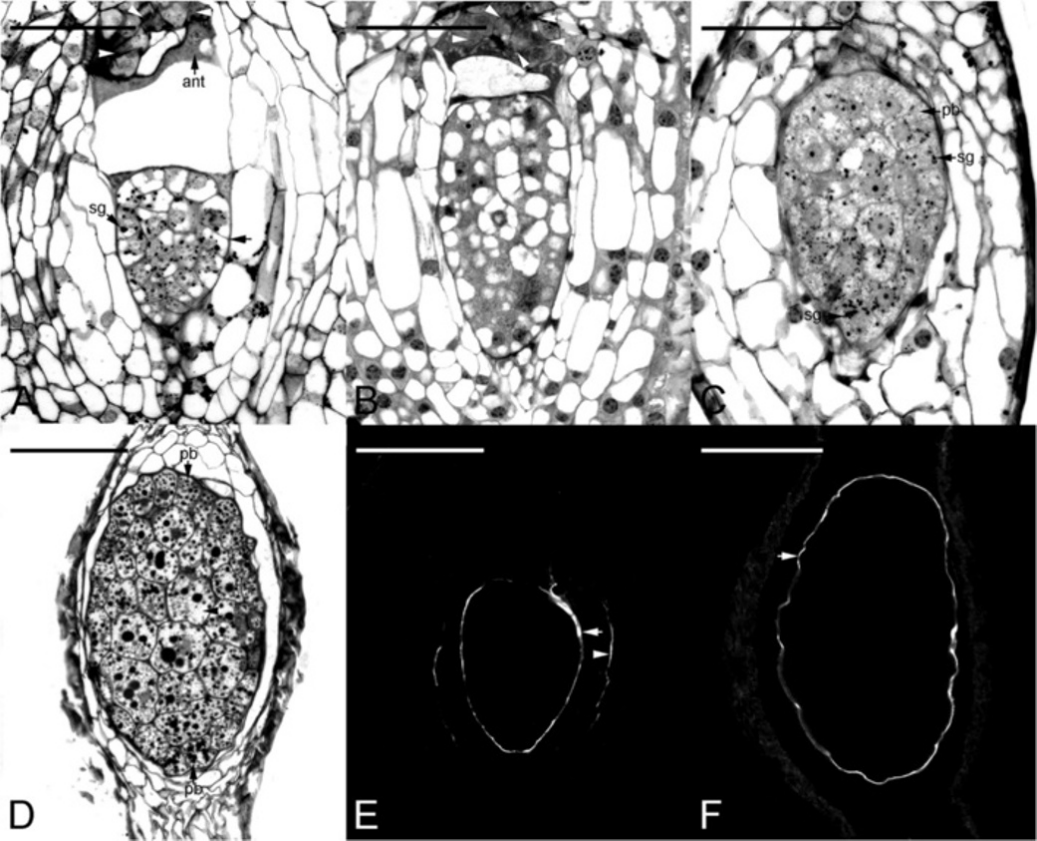
**Late embryo development of** ***Cyrtosia javanica*** **. (A)** Light micrograph showing a longitudinal section through an early globular embryo with a differentiating protoderm layer (arrowhead). Several starch grains could be observed throughout the embryo cells after the periodic acid-Schiff’s (PAS) reaction. The chalazal accessory cells are distinguished from the adjacent cells of seed coat by their densely stained cytoplasm (arrowheads). **(B)** Light micrograph showing a further elongation of a globular embryo by the increase of cell number and the vacuolation. The chalazal accessory cells are distinguished from the adjacent cells of seed coat by their densely stained cytoplasm (arrowheads). **(C)** Light micrograph showing a maturing embryo. At this stage, there are a few starch grains persisted and they tend to congregate around the nucleus; numerous tiny protein bodies have accumulated within the embryo proper. **(D)** Light micrograph showing a longitudinal section through a mature seed. Many tiny protein bodies can be found within the embryo proper cells after the protein staining with amido black 10B. In this preparation, the lipid bodies are not preserved, the spaces (arrowhead) between the protein bodies are occupied by storage lipid bodies. **(E)** Nile red staining fluorescence micrograph of an orchid seed at the stage similar to Figure 3A. At the globular stage, the fluorescence outline is first detected in the surface of the embryo proper (arrow) and the outermost wall of the outmost layer of the inner seed coat (arrowhead). **(F)** Nile red staining fluorescence micrograph of a mature seed at the stage similar to Figure 3D. At maturity, the inner seed coat has compressed into a thin layer and attached the embryo tightly, and the surface of the embryo proper fluoresces brightly (arrow). Abbreviations: ant = antipodal cells; pb = protein body; sg = starch grain. Scale bar = 100 μm.

After fertilization, the zygote of *C. javanica* had an ovoid shape that was highly polarized (Figure 2A). The nucleus and most cytoplasm located toward the chalazal end, while the micropylar end was highly vacuolated. The endosperm failed to develop in this species, and the endosperm nuclei were eventually absorbed by the embryo during the early stages of embryo development (Figure 2B, C and D). The first division of the zygote was transverse and gave rise to a two-celled embryo with similar cell sizes (Figure 2B). Soon after, the embryo cells divided further and resulted in the formation of a proembryo (Figure 2C). At the proembryo stage, the antipodal cells had the densely stained cytoplasm and attached to the accessory cells in the chalazal end (Figure 2C and D). During the early stages of embryo development, cell divisions within the embryo proper were active and resulted in the formation of a spheroidic embryo proper (Figure 2D and E). The anticlinal division from the surface layer resulted in the formation of the protoderm (Figure 3A). At the globular stage, additional cell divisions occurred in the inner cell tiers contributing to the growth of embryo proper, and the chalazal accessory cells could be easily recognized by their densely stained cytoplasms (Figure 3A and B). At maturity, the embryo was only eight cells long and five to six cells wide (Figure 3D). Throughout the embryo development, the embryo of this species lacked a structurally defined suspensor (Figure 3A-D).

### Seed coat

**Figure 4:**
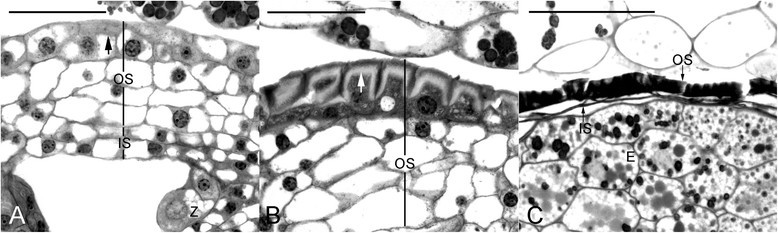
**The seed coat development of** ***Cyrtosia javanica*** **. (A)** The seed coat is composed of the inner (two cells thick) and outer (four cells thick) seed coats. At the zygote stage, the cell wall of the outer most layer of outer seed coat is thickening (arrow). **(B)** At the early globular stage, the cell wall of the outer most layer of outer seed coat has thickened (arrow). **(C)** At maturity, both inner and outer seed coats have dehydrated and compressed that enveloped the embryo (E) tightly. Abbreviations: IS = inner seed coat; OS = outer seed coat; Z = zygote. Scale bar = 100 μm.

The seed of *C. javanica* was relatively large (6 to 8 mm in length) in the orchid family (Figure 1C and D). At seed maturity, the embryo was enveloped by two layers of seed coat: the inner seed coat was thin, while the outer seed coat was thickened and sclerified (Figure 4C). The inner seed coat was two cells thick (Figure 4A). During the early stages of embryo development, the cells of the inner seed coat were highly vacuolated (Figure 2A-C). As the seeds approached maturity, the inner seed coat gradually crushed (Figure 3C), and finally became a thin layer at maturity (Figure 4C). In contrast to the inner seed coat, the outer seed coat was four to five cells thick (Figure 4A). During the early stages of embryo development, the cytoplasm of the outermost cell layer was denser as compared to the other cell layers of the outer seed coat (Figure 4A). At the globular stage, the outer and lateral cell walls of the outermost cell layer of the outer seed coat became thickened (Figure 4B). At maturity, all the cell layers of the outer seed coat had compressed, resulting in the formation of a hard sclerified outer seed coat (Figures 3D and 4C).

### Histochemical investigations

At the proembryo stage, the cytoplasm reacted strongly with amido black 10B, a protein stain (Figure 2C and D). However, no distinct protein body-like structures were found within the cytoplasm of the proembryo. At the early globular stage, starch grains began to appear within the embryo proper (Figure 3A). After the cells had ceased to divide, a few small protein bodies could be observed at the basal part of embryo proper (Figure 3C). As the seeds approached maturity, the large vacuoles had broken down and more protein bodies had accumulated within the cytoplasm of the embryo proper. Together, lipid bodies began to accumulate within the cytoplasm. At seed maturity, protein and lipid bodies were the major storage products within the embryo proper (Figure 3D).

In mature seeds, the thickened cell walls of the outermost layer of the outer seed coat stained greenish blue with the TBO stain, indicating the presence of phenolic compounds in the wall (Figure 4B and C). When stained with Nile red at the early globular stage, only the lateral walls of the outermost layer of inner seed coat reacted weakly (Figure 3E), suggesting the accumulation of cuticular substance or the presence of secondary walls (TBO stain) at the lateral walls. Besides, Nile red staining indicated the presence of cuticular material in the surface wall of embryo proper (Figure 3E and F). Throughout the seed development and maturation, the cell layers of outer seed coat reacted negatively to the Nile red stain (Figure 3E and F).

## Discussion

Orchid seeds are characterized by their minute size and light weight (Dressler [1993]). Despite their tiny size, a considerable variation of seed morphology could be observed among orchid species (Arditti and Ghani [2000]). As compared to the seed size of most orchids, the members of subfamily Vanilloideae, e.g. *Cyrtosia*, *Galeola* and *Vanilla*, have relatively large seeds (Clements and Molvray [1999]). The present study on histology and histochemistry of developing seeds of *C. javanica* revealed some remarkable features: the first, without a structurally defined suspensor during embryogenesis; the second, the hard seed coat with the lignification in the outermost cell layer of the outer seedcoat.

The suspensor plays an important role during the embryo development that facilitates nutrient movement from the maternal tissues to the embryo proper (Yeung and Meink [1993]). Generally, the first division of the zygote was asymmetrically, producing two daughter cells of different sizes and fates. The smaller terminal cell results in the formation of the embryo proper, while the larger basal cell gives rise to the suspensor and also the basal part of the embryo (Goldberg et al. [1994]). In *C. javanica*, the first cell division of zygote produced a terminal cell and basal cell of the same size. Subsequently, the embryo cells further divided and resulted in the formation of a spheroidic embryo proper without a structurally defined suspensor (Figure 2C-F). In orchid family, the suspensor morphology is diverse (Swamy [1949]). Some species are without a structurally defined suspensor as in *Spiranthes* (Clements [1999]); some species have filamentous cells as in *Phalaenopsis* (Lee et al. [2008]), or the haustoria-like structure as in *Habenaria* (Swamy [1949]). In Nun orchid, the suspensor is the main site for nutrient transport during embryo development (Lee and Yeung [2010]). For those orchids without a structurally defined suspensor such as *Cyrtosia* here and *Spiranthes* (Clements [1999]), we may propose that the developing embryo proper could acquire the nutrients from adjacent tissues. In *C. javanica*, it is worthy to note that the chalazal accessory cells are distinguished from the adjacent cells of seed coat by their densely stained cytoplasm (Figure 3A and B). These chalazal accessory cells look similar to the specialized chalazal cyst in the seeds of *Arabidopsis* and *Lepidium* in Brassicaceae (Nguyen et al. [2000]; Brown et al. [2004]). Moreover, both the chalazal and micropylar ends of seed coat tissues reacted negatively to the Nile red staining, indicating no apoplastic barriers present during embryo development (Figure 3E). It is not clear that the chalazal accessory cells may perhaps function as the chalazal cyst for transport of metabolites into the developing seed. Further ultrastructural and immunohistochemical studies on the chalazal accessory cells could provide insights of their function during embryo development.

The seed coat protects developing embryos from drying and mechanical injury that is derived from the integument tissue (Bewley and Black [1994]). In the mature seeds of orchids, the seed coat is usually composed of one (epiphytic orchids, e.g. *Phalaenopsis*) or two (terrestrial orchids, e.g. *Cypripedium*) thin layers (Yam et al. [2002]; Lee et al. [2005]; Lee et al. [2008]). In *C. javanica*, two layers of the seed coat, i.e. the thickened outer seed coat and the thin inner seed coat are present as the seeds matured (Figure 4C). It is worthy to note that the outer seed coat is derived from four layers of cells, and the outermost cell layer has become thickened at the early globular stage (Figure 4A and B). Mature seeds of several vanilloid species are very hard because the walls of seed coats are heavily thickened with perhaps the lignin polymers (Cameron and Chase [1998]; Nishimura and Yukawa [2010]). In *Vanilla* (a close related genus of *Cyrtosia*), the walls of seed coats heavily thickened with the caffeyl alcohol derived lignin polymers are recently identified by nuclear magnetic resonance spectroscopy (Chen et al. [2012]). In this study, the histochemical staining of the outer seed coat reveals that the cell walls contain phenolic substances, indicating the accumulation of lignin in the thicken seed coat (Figure 4C). The sclerotization in the outer seedcoat has been observed in some basal orchid groups, such as *Apostasia* (the subfamily Apostasioideae) and *Vanilla* (the subfamily Vanilloideae), suggesting a plesiomorphic character of the seed in Orchidaceae (Nishimura and Tamura [1993]; Nishimura and Yukawa [2010]). Several vanilloid orchids, such as *Cyrtosia*, *Erythrorchis*, *Galeola* and *Vanilla* are different from most orchids by having fleshy fruits. As the fruits of *Cyrtosia* and *Vanilla* matured, they start to turn red (Figure 1B) or have fragrances to attract the visiting of bats or birds (Soto Arenas and Dressler [2010]). Therefore, it is suggested that the lignification of seed coat could protect the embryo survival when pass through the alimentary canal of animals (Rodolphe et al. [2011]). *C. javanica* is a fully mycoheterotrophic orchid species, and the seeds mature just within a month (according to Nishimura and Yukawa ([2010]), the seeds of *Vanilla* require six months for maturation). The mechanisms that allow for formation of lignin polymers within a short period remain to be investigated.

## Conclusions

In *C. javanica*, the detail structural and histochemical studies of embryology such as embryo development and the formation of seed coat were investigated. The large and heavy seeds embedded in fresh fruits may adapt to the dispersal strategy. In our preliminary experiments, the seed germination of *C. javanica* is difficult. The hard seeds with lignified outer seed coat could provide a rigid protection during seed dispersal but also cause coat-imposed dormancy. This study provides insights in the seed coat structure and the hints of seed treatment methods.

## Authors’ original submitted files for images

Below are the links to the authors’ original submitted files for images. Authors’ original file for figure 1 Authors’ original file for figure 2 Authors’ original file for figure 3 Authors’ original file for figure 4
