# Supplementary figures and images for: The seed development of a mycoheterotrophic orchid, Cyrtosia javanica Blume
Source: Bot Stud. 2014 May 30;55:44. doi: 10.1186/s40529-014-0044-8 (PMC5430362; doi:10.1186/s40529-014-0044-8)

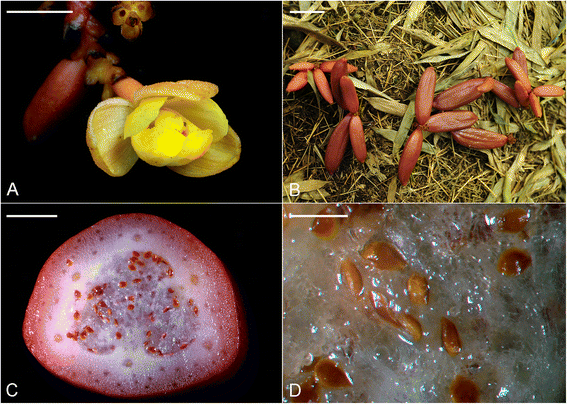

Supplement: Supplementary file 1 — Authors’ original file for figure 1 [file 40529_2014_44_MOESM1_ESM.gif]

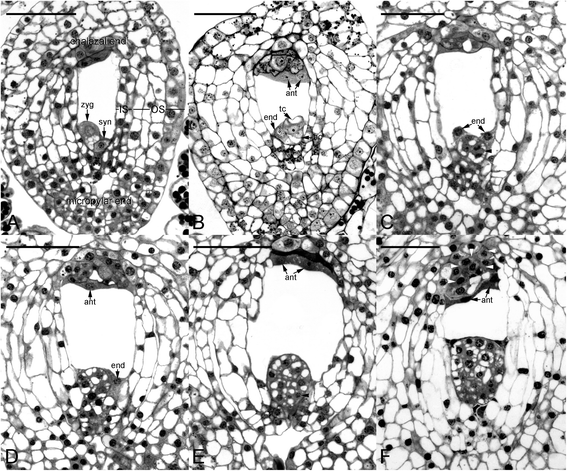

Supplement: Supplementary file 2 — Authors’ original file for figure 2 [file 40529_2014_44_MOESM2_ESM.gif]

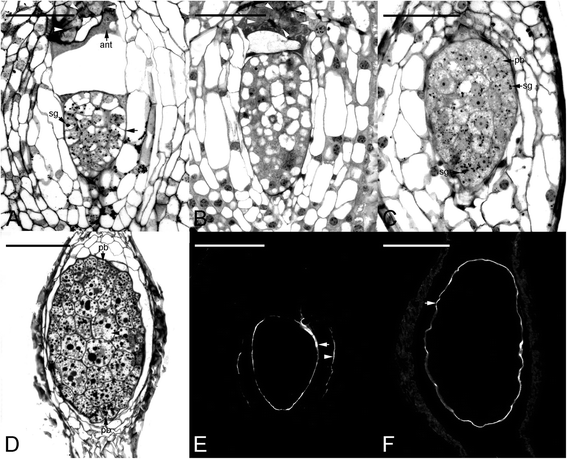

Supplement: Supplementary file 3 — Authors’ original file for figure 3 [file 40529_2014_44_MOESM3_ESM.gif]

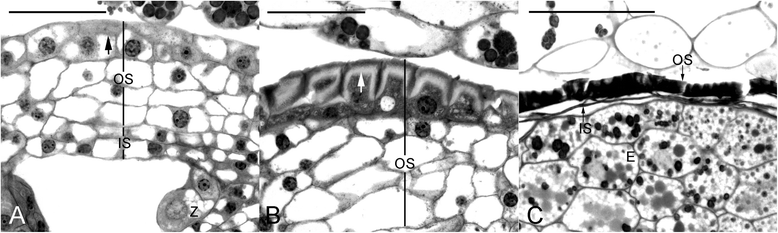

Supplement: Supplementary file 4 — Authors’ original file for figure 4 [file 40529_2014_44_MOESM4_ESM.gif]
